# Editorial: Role of nanocarriers and non-coding RNAs in solid malignancies

**DOI:** 10.3389/fonc.2025.1737094

**Published:** 2025-12-12

**Authors:** Rana A. Youness, Sherif Ashraf Fahmy, Eleni K. Efthimiadou, Maria Braoudaki, Gowhar Rashid

**Affiliations:** 1 Molecular Genetics and Biochemistry Department, Molecular Genetics Research Team (MGRT), Faculty of Biotechnology, German International University (GIU), Cairo, Egypt; 2 Department of Pharmacy, Institute of Pharmaceutics and Biopharmaceutics, Marburg University, Marburg, Germany; 3 Inorganic Chemistry Laboratory, Department of Chemistry, National and Kapodistrian University of Athens, Zografou, Greece; 4 School of Health, Medicine and Life Sciences, University of Hertfordshire, Hatfield, United Kingdom; 5 Department of Clinical Biochemistry, Sher-i-Kashmir Institute of Medical Sciences (SKIMS), Srinagar, J&K, India

**Keywords:** nanocarrier, non-code RNA, lncRNAs, miRNAs, cancer, oncology

The fight against solid malignancies is characterized by two persistent challenges: tumor heterogeneity and the formidable task of drug delivery. Conventional therapeutics often fail due to systemic toxicity, poor tumor accumulation, and inability to penetrate the dense tumor microenvironment (TME). However, a potent synergy between nanocarriers and non-coding RNAs (ncRNAs) is now poised to redefine therapeutic precision.

The realization that over 98% of the human genome does not code for proteins has shifted our focus to the regulatory landscape governed by non-coding RNAs (ncRNAs), particularly microRNAs (miRNAs) and long non-coding RNAs (lncRNAs). These molecules are not merely bystanders; they are master regulators of gene expression, controlling pathways involved in cell proliferation, apoptosis, metastasis, and, critically, drug resistance.

In solid malignancies, ncRNAs are often dysregulated, acting as either potent oncogenes (onco-ncRNAs) or tumor suppressors. Targeting these regulatory hubs, for instance, by using siRNAs to silence an oncogenic lncRNA or restoring a downregulated tumor-suppressing miRNA, offers an unparalleled level of therapeutic specificity. This strategy moves beyond simply blocking a single protein to fundamentally reprogramming the malignant phenotype.

The challenge for ncRNA-based drugs is maintaining stability and ensuring effective delivery. Naked nucleic acids are rapidly degraded by nucleases in the circulation, exhibit low cellular uptake, and cannot cross key biological barriers (like the blood-brain barrier for glioblastoma). This is where nanocarriers become indispensable.

Nanotechnology enables the development of highly engineered vehicles, including liposomes, polymeric micelles, and dendrimers, which serve as protective molecular couriers. Their sub-100 nm size allows them to maintain the Enhanced Permeability and Retention (ERP) effect, passively accumulating in the leaky vasculature of solid tumors. Furthermore, they can be functionalized with targeting ligands (e.g., peptides or antibodies) for active, receptor-mediated uptake by cancer cells, drastically improving the therapeutic index and sparing healthy tissue.

The future of precision oncology in solid malignancies rests heavily on the continued synergy of these two fields. Research must prioritize developing next-generation nanocarriers optimized for TME penetration and efficient intracellular release. By successfully bridging the gap between potent molecular targets and sophisticated delivery systems, we move closer to the era where cancer therapy is truly a programmable, precise intervention.

This Research Topic further cements the ncRNA field’s dual role, extending beyond therapy into crucial diagnostic applications. The systematic review by Assal et al. is another notable contribution, highlighting the therapeutic potential of targeting specific long non-coding RNAs in genitourinary malignancies. This comprehensive review focuses on Maternally Expressed Gene 3 (MEG3), which is frequently dysregulated in various tumors. Specifically, the authors unravel the multifaceted mechanistic roles of MEG3 in genitourinary malignancies, detailing its influence on key cancer processes like cellular proliferation, apoptosis, invasion, and metastasis. By mapping these intricate molecular pathways, the review establishes MEG3 as a critical, dual-purpose player, both a highly promising biomarker for improved diagnosis and a novel therapeutic target, further emphasizing the shift towards molecular reprogramming as a core strategy in solid tumor oncology.

Another interesting article published in the Research Topic by Abdelbary et al. highlights the necessity of understanding the influence of ncRNAs on the tumor microenvironment. This point was addressed by a key functional study that investigates the role of miR-216a-3p in Hepatocellular Carcinoma (HCC). Recognizing the reported reduction in Natural Killer (NK) cell cytotoxicity in HCC patients, this research found that miR-216-3p is highly expressed in NK cells from these patients. Mechanistically, forcing the expression of this miRNA in healthy NK cells severely impaired their cytotoxic activity, evidenced by a reduced release of effector molecules like TNF-α, IFN-γ and Granzyme B. Furthermore, the study links this immunosuppressive effect to the FOXO-signaling pathway, demonstrating that miR-216a-3p downregulates the critical NK cell maturation factor FOXO-1. This work robustly identifies miR-216a-3p as a negative regulator of NK cell cytotoxicity, positioning it as a novel target for immune-modulating cancer therapies.

Another systematic review article by Sun et al. on the FOXO3a-miRNA regulatory axis offers a detailed examination of the complexity of tumor suppression. FOXO3a, a key forkhead box transcription factor, is known to inhibit tumor growth; however, its expression is also regulated by miRNA targeting. Analyzing five tumor types, the review categorized miRNA interactions into distinct functional groups: synergistic (where miRNAs and FOXO3a collaborate to inhibit cancer growth, as seen in breast cancer and HCC) and antagonistic (where miRNAs suppress FOXO3a activity, promoting tumor development). Crucially, the review highlights that FOXO3a’s function is context-dependent; in certain combined interactions with lncRNAs and specific miRNAs (like miR-485-5p), it surprisingly switches to promote tumor progression. These diverse outcomes are linked to several major oncogenic pathways, including PI3K/AKT and Wnt/β-catenin pathways, underscoring the potential of FOXO3a-miRNA interactions as a crucial point for therapeutic intervention.

Finally, an interesting review article by Elsori et al. addresses the urgent need for novel, disease-modifying therapies for Alzheimer’s disease (AD) and brain tumors. It spotlights nanomaterials, particularly carbon nanotubes (CNTs), as a promising dual therapeutic arsenal. The unique properties of CNTs, including their ability to easily cross the blood-brain barrier (BBB) and their chemical adaptability for targeted functionalization, make them ideal candidates for specific drug delivery and neuroprotection. By exploring the mechanisms by which CNTs penetrate cell membranes and deliver medication, this review aims to pave the way for effective and innovative treatment strategies for these challenging neurological conditions.

